# Nanotopographical Features of Polymeric Nanocomposite Scaffolds for Tissue Engineering and Regenerative Medicine: A Review

**DOI:** 10.3390/biomimetics10050317

**Published:** 2025-05-15

**Authors:** Kannan Badri Narayanan

**Affiliations:** 1 School of Chemical Engineering, Yeungnam University, 280 Daehak-Ro, Gyeongsan, Gyeongbuk 38541, Republic of Korea; okbadri@gmail.com or okbadri@yu.ac.kr; 2 Research Institute of Cell Culture, Yeungnam University, 280 Daehak-Ro, Gyeongsan, Gyeongbuk 38541, Republic of Korea

**Keywords:** nanotopography, nanomaterials, nanocomposites, polymers, natural/synthetic scaffolds, biomaterials, tissue engineering

## Abstract

Nanotopography refers to the intricate surface characteristics of materials at the sub-micron (<1000 nm) and nanometer (<100 nm) scales. These topographical surface features significantly influence the physical, chemical, and biological properties of biomaterials, affecting their interactions with cells and surrounding tissues. The development of nanostructured surfaces of polymeric nanocomposites has garnered increasing attention in the fields of tissue engineering and regenerative medicine due to their ability to modulate cellular responses and enhance tissue regeneration. Various top-down and bottom-up techniques, including nanolithography, etching, deposition, laser ablation, template-assisted synthesis, and nanografting techniques, are employed to create structured surfaces on biomaterials. Additionally, nanotopographies can be fabricated using polymeric nanocomposites, with or without the integration of organic and inorganic nanomaterials, through advanced methods such as using electrospinning, layer-by-layer (LbL) assembly, sol–gel processing, in situ polymerization, 3D printing, template-assisted methods, and spin coating. The surface topography of polymeric nanocomposite scaffolds can be tailored through the incorporation of organic nanomaterials (e.g., chitosan, dextran, alginate, collagen, polydopamine, cellulose, polypyrrole) and inorganic nanomaterials (e.g., silver, gold, titania, silica, zirconia, iron oxide). The choice of fabrication technique depends on the desired surface features, material properties, and specific biomedical applications. Nanotopographical modifications on biomaterials’ surface play a crucial role in regulating cell behavior, including adhesion, proliferation, differentiation, and migration, which are critical for tissue engineering and repair. For effective tissue regeneration, it is imperative that scaffolds closely mimic the native extracellular matrix (ECM), providing a mechanical framework and topographical cues that replicate matrix elasticity and nanoscale surface features. This ECM biomimicry is vital for responding to biochemical signaling cues, orchestrating cellular functions, metabolic processes, and subsequent tissue organization. The integration of nanotopography within scaffold matrices has emerged as a pivotal regulator in the development of next-generation biomaterials designed to regulate cellular responses for enhanced tissue repair and organization. Additionally, these scaffolds with specific surface topographies, such as grooves (linear channels that guide cell alignment), pillars (protrusions), holes/pits/dots (depressions), fibrous structures (mimicking ECM fibers), and tubular arrays (array of tubular structures), are crucial for regulating cell behavior and promoting tissue repair. This review presents recent advances in the fabrication methodologies used to engineer nanotopographical microenvironments in polymeric nanocomposite tissue scaffolds through the incorporation of nanomaterials and biomolecular functionalization. Furthermore, it discusses how these modifications influence cellular interactions and tissue regeneration. Finally, the review highlights the challenges and future perspectives in nanomaterial-mediated fabrication of nanotopographical polymeric scaffolds for tissue engineering and regenerative medicine.

## 1. Introduction

Tissue scaffolds play a vital role both in vitro and in vivo for recapitulating the natural three-dimensional (3D) tissue development process, thereby enabling cells to create their own microenvironments [1]. These scaffolds offer a 3D matrix that supports cell proliferation, migration, and matrix production, ultimately leading to the formation of functional tissues with specific shapes. They also provide structural integrity for developing tissues and facilitate the integration of biological or mechanical cues to enhance tissue formation. The design of scaffold properties, both biological and mechanical, can be tailored to specific applications, incorporating signals that promote cell proliferation and differentiation of specific cell types [2]. Moreover, the surface topography of 3D tissue scaffolds significantly influences cell behavior, including adhesion, proliferation, migration, orientation, elongation, differentiation, and apoptosis, with key factors being surface patterns, roughness, and porosity [3,4].

Mammalian cells respond to various cues from their microenvironment, including surface topography, chemistry, hydrophobicity, surface energy, and mechanical properties. These biophysical and biochemical signals at the cell–substrate interface are critical considerations in directing cellular behavior such as adhesion, proliferation, migration, and differentiation in tissue engineering and regenerative medicine [5]. In the context of tissue engineering and regenerative medicine, the interaction between cells and biomaterial surfaces is fundamental to the efficacy of scaffold-based approaches. While natural biomaterials (e.g., collagen, gelatin, and chitosan) inherently support biological interactions, they lack the mechanical strength, degradation control, and flexibility required for clinical applications. In contrast, synthetic biomaterials can be engineered to possess tunable mechanical and structural properties, and their intrinsic bio-inertness can be overcome by functionalization with bioactive motifs to enhance cellular attachment and activity [6]. The development of wound dressings using the electrospun polycaprolactone (PCL) incorporated with titanium dioxide (TiO_2_) nanopowder was enhanced by coating the scaffold with the natural polymer collagen. This modification increased the hydrophilicity of the PCL nanofibers, promoting cell adhesion and proliferation [7]. In another instance, collagen-coated ostholamide (OSA)-incoporated polyhydroxybutyrate (PHB) and gelatin (GEL) electrospun nanofiber scaffolds (OSA-PHB-GEL) demonstrated excellent cytocompatibility, promoting NIH- 3T3 fibroblast cell proliferation and enhancing wound healing efficacy in Wistar rats [8].

Designing biomaterials with optimal physical, mechanical, and biological properties is essential for the formation of functional tissues. The key physicochemical properties of biomaterials, such as surface topography, charge, and mechanical stiffness, greatly influence the initial adhesion of cells and subsequent cell–biomaterial interactions [9]. Generally, the biological properties of biomaterials are largely determined by the ability of cells to interact with and attach to their surfaces. This interaction is primarily dependent on the availability of proteins or cellular components for adhesion, overcoming the bio-inertness of scaffolds and functioning as cell-friendly surfaces [10]. Surface modifications, such as incorporating extracellular matrix (ECM) proteins with fibronectin-binding motifs—such as arginine–glycine–aspartic acid (RGD), RGD–serine (RGDS), leucine–aspartic acid–valine (LDV), arginine–glutamic acid–aspartic acid–valine (REDV), and lysine–glycine–aspartic acid (KGD) sequences—enhance cell adhesion, migration, and signaling processes for tissue regeneration. However, these functionalization processes through conjugation procedures can be expensive and involve toxic chemicals, posing potential harm to cells. To address these challenges, fabricating biomaterial surfaces with nanotopography has emerged as a promising strategy to enhance cell adhesion and behavior. Nanocomposite technology has also been shown to improve the mechanical and biological properties of biomaterials through topographical modification by influencing their surface roughness [11].

The advent of nanotechnology has integrated multiple scientific disciplines, opening avenues for developing novel therapeutics, diagnostics, and improved theranostics. This has fueled interest in fabricating biomaterials with nanoscale surface topography for tissue engineering and regenerative medicine applications [12]. Cells are naturally surrounded by a three-dimensional (3D) microenvironment that includes micro- and nano-scaled ECM components, such as globular and fibrillar structures [13,14]. For example, collagen molecules in the ECM are approximately 300 nm long and 1.5 nm wide, forming micrometer-sized fibrils [15]. The intensity of the host cell and immune response is primarily influenced by the biomaterial composition, as well as the porosity, hydrophobicity, topography, and biodegradability of the scaffold. These factors collectively regulate the recruitment and activation of cellular mediators following implantation, which ultimately impact tissue integration and healing [16]. Earlier studies, such as those by Woo et al. [17], demonstrated that synthetic nanofibrous poly(L-lactic acid) (PLLA) scaffolds mimicking the 3D structure of ECM collagen fibers significantly enhance ECM protein adsorption, leading to increased (>1.7 times) osteoblast attachment for tissue engineering. Such intricate ECM provides control over cellular processes and signaling pathways necessary for cell polarization, migration, and 3D tissue organization through contact guidance [18,19,20]. Synthetic nanofabricated 3D biomaterials, which are analogous to native ECM have been shown to influence cell morphology, alignment, adhesion, proliferation, migration, differentiation, and tissue organization [21,22,23].

**Figure 1:**
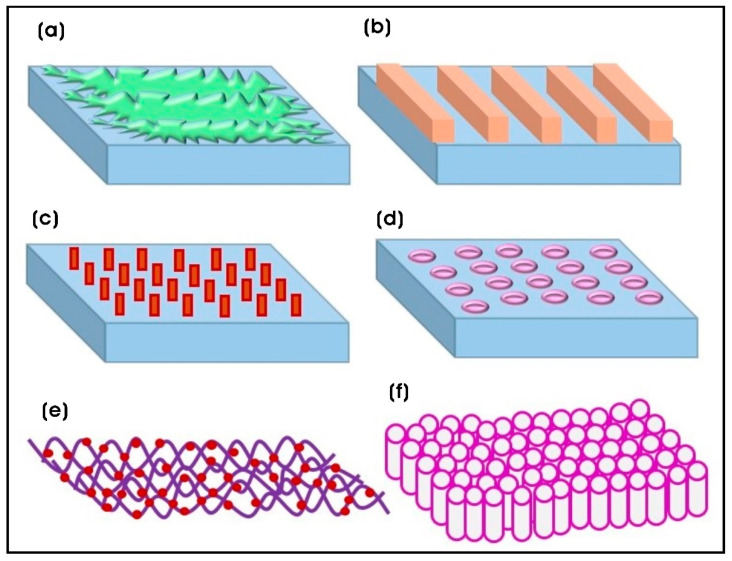
Different nanopatterned surfaces for tissue engineering and regenerative medicine applications. (**a**) Surface roughness; (**b**) grooves; (**c**) pillars; (**d**) holes/pits/dots; (**e**) fibrous; (**f**) tubular array topographies.

The properties of biomaterials are strongly influenced by the characteristics of the substrate, which play a crucial role in modulating tissue–material interactions, and ultimately, in promoting effective tissue regeneration. Nanostructures can be integrated onto biomaterial surfaces using diverse top-down and/or bottom-up techniques, including nanolithography, etching, deposition, laser ablation, template-assisted synthesis, and nanografting [24]. Similarly, polymeric nanocomposite biomaterials featuring surface-coated or incorporated nanomaterials, such as organic and/or inorganic nanomaterials, generate nanotopographical surfaces, thereby imparting scaffold-specific functions to enhance cellular interactions and behavior for tissue engineering and other biomedical applications (Figure 1). Incorporating nanomaterials containing growth factors, cytokines, and therapeutic agents enables sustained or controlled release and supports cell and tissue development [25]. Furthermore, nanoscale structures improve the mechanical properties of scaffolds by offering a large surface-to-volume ratio, enhancing nutrient transport and gas diffusion, which positively influences cellular metabolism and interactions between cells and biomaterials [26]. This review highlights recent advances in the fabrication of biomaterials with tailored surface properties using various techniques that integrate sub-micron (<1000 nm) and nanoscale (<100 nm) topographical cues onto polymeric nanocomposites. These techniques involve the incorporation of both organic and inorganic nanomaterials, as well as biomolecule functionalization, and demonstrate their influence on interactions with different mammalian cells for applications in tissue engineering and regenerative medicine.

## 2. Overview of Polymeric Nanocomposite Scaffolds

Polymeric nanocomposites are materials composed of a polymer matrix and a nanoscale filler or reinforcement phase, where at least one dimension of the dispersed phase is in the nanometer range (typically <100 nm). These nanostructured fillers, such as nanoclays, nanofilms, nanoflakes, nanoparticles, nanotubes, and nanofibers, are incorporated to enhance the physicochemical, thermal, and mechanical properties of the polymer matrix [27]. These polymeric nanocomposites can incorporate organic, inorganic, or hybrid materials. These nanomaterials can be categorized into several classes based on their composition: carbon-based nanomaterials such as graphene, graphene oxide, carbon nanotubes, and fullerenes [28]; inorganic nanoparticles (metal and metal oxide) such as silica, titania, ceria, zinc oxide, iron oxide, copper, silver, and gold; inorganic clay minerals such as montmorillonite, kaolinite, halloysite, and bentonite; natural mineral such as whitlockite; ceramic nanomaterials such as hydroxyapatite, zirconia, and bioglass; and natural/synthetic polymeric nanoparticles such as collagen, cellulose, chitosan, gelatin, alginate, polydopamine (PDA), poly(ɛ-caprolactone) (PCL), poly(vinyl alcohol) (PVA), poly(ethylene glycol) (PEG), poly-L-lactic acid (PLLA), poly-L/D-lactide (PLDLA), and poly(lactic-co-glycolic acid) (PLGA) [29,30]. These nanomaterials can serve as nanofillers with different dimensionalities: 0D nanofillers such as nanoclays, nanodiamonds, and fullerenes; 1D nanofillers such as nanotubes and nanofibers; 2D nanofillers such as nanofilms and nanoflakes; and 3D nanofillers such as nanoparticles and nanostructure. Each type exhibits distinct functions, shapes, and structures that contribute to variations in surface topography, influencing the properties of polymeric nanocomposites [31,32].

Polymeric nanocomposite scaffolds are fabricated using techniques such as electrospinning, 3D printing, freeze-drying, and solvent casting, allowing for precise control over scaffold morphology and functional properties. These scaffolds undergo crosslinking through various methods, including physical crosslinking (e.g., ionic gelation, hydrogen bonding, hydrophobic interactions, and thermosensitive gelation), chemical crosslinking (e.g., free radical polymerization, covalent conjugation, and enzymatic conjugation), and photo-crosslinking [33]. For applications in tissue engineering and regenerative medicine, polymeric nanocomposite scaffolds must be biocompatible, biodegradable, non-toxic, and sustainable. Additionally, scaffolds must provide interconnected porosity and suitable pore morphology, ensuring enhanced mechanical properties that facilitate cell–biomaterial interactions for effective integration with host tissue.

## 3. Cellular Responses to Nanotopography of Polymeric Nanocomposite Scaffolds

Nanoscale topography plays a crucial role in directing cell behavior through contact guidance, a process in which cells respond to the nanoscale features of the extracellular matrix (ECM) for various cellular processes and functions. The ECM exhibits submicronic (<1000 nm) and nanoscale (<100 nm) topographies that regulate cell adhesion, migration, and differentiation [34]. Collagen fibers (100–500 nm) form fibrillar networks [35], while fibronectin (200–500 nm) and elastin (500–1000 nm) contribute to structural integrity [36,37]. Nanotopographies include collagen triple helices (~1.5 nm), proteoglycans (2–50 nm), and basement membrane nanostructures (10–50 nm), which influence integrin-mediated signaling [38,39,40,41,42]. These features guide stem cell fate, focal adhesion formation, and mechanotransduction [43]. Mimicking ECM topographies enhances biomaterial integration and tissue regeneration. Membrane-bound receptors such as integrins interact with ECM proteins at these nanoscale dimensions, forming focal adhesions essential for cell adhesion, migration, and differentiation [44]. The geometry and size of nanotopographical features significantly impact focal adhesion formation, with smaller nanostructures (e.g., nanopillars ~200 nm) enhancing adhesion due to increased interaction with cell surface receptors. Cells generally adhere more effectively to substrates featuring nanotopographical patterns compared to flat substrates, thereby improving adhesion-mediated signaling pathways [39,42,45,46]. Nanotopographical cues influence cell functions by modulating mechanotransduction pathways. Cells sense not only topographical features but also substrate rigidity and chemical composition, which regulate cellular responses [44].

Polymeric nanocomposites, though often containing randomly arranged nanoscale components, can be engineered with controlled nanotopographies with desired patterning using nanofabrication techniques. These approaches allow the precise application of ECM-like surface features critical for effective cell adhesion and tissue regeneration, with responses governed by cell type, feature size, and geometry [21,47]. In addition to nanotopography, surface chemistry also plays a pivotal role in cellular interactions. Coating materials with ECM proteins such as collagen I, fibronectin, elastin, and laminin provide enhanced cell adhesion by providing binding sites, thereby enhancing biointerfacial interactions [48]. For instance, the basement membrane of the corneal epithelium in rhesus macaques exhibits nanoscale features, including elevations (76–379 nm) and pores (22–216 nm), with an average pore spacing of 87 nm. These 3D nanotopographies increase the surface area of the basement membrane by up to 400%, which significantly influences the cell–surface interactions, thereby regulating the tissue growth [38].

The size and morphology of nanomaterials also affect cell adhesion and proliferation. Smaller nanoparticles embedded in scaffolds enhance cell interactions, whereas larger particles hinder cell adhesion [49]. For instance, spherical hydroxyapatite nanoparticles (nHAp) promoted higher proliferation of L929 fibroblasts compared to needle-shaped HA nanoparticles [50]. Similarly, incorporating 10–30% nanohydroxyapatite (nHAp) into poly(lactic acid) (PLA) microspheres improved the physicochemical and biological properties of the microspheres, increasing the surface hydrophilicity and roughness, and promoting the adhesion and proliferation of rat mesenchymal stem cells (rMSCs) for tissue repair. Additionally, these composite microspheres exhibited significant osteogenic differentiation of rMSCs [51]. As nano-HAP is a major constituent of natural bone, it is well known for its osteoconductive properties. Chi et al. [52] demonstrated that 3D-printed PLA scaffolds coated with polydopamine (PDA) (PLA@PDA) and immobilized nano-HAP promoted osteoblastic differentiation and mineralization, making them suitable for bone tissue engineering. Likewise, physically crosslinked nanocomposite films of poly(ethylene oxide) (PEO) with layered silicate nanoparticles (laponite) positively influenced fibroblast adhesion, which was absent in pure PEO. Higher laponite concentrations in the composite significantly supported cell proliferation and spreading. These polymeric nanocomposites, with diverse nanotopographical features, hold promise for the development of novel biomaterials designed to regulate cellular adhesion and functions for various biomedical applications [53]. Thus, cellular responses to nanotopographies are influenced by multiple factors, including material composition, nanomaterial/nanofiller characteristics, biomolecule functionalization, and surface geometry. Understanding these interactions is crucial for developing biomaterials that effectively support tissue engineering and regenerative medicine applications. Advances in nanofabrication techniques continue to provide insights into optimizing biomaterial surfaces to enhance cell function and tissue regeneration.

## 4. Fabrication Techniques of Polymeric Nanocomposite Scaffolds with Nanotopographies

**Figure 2:**
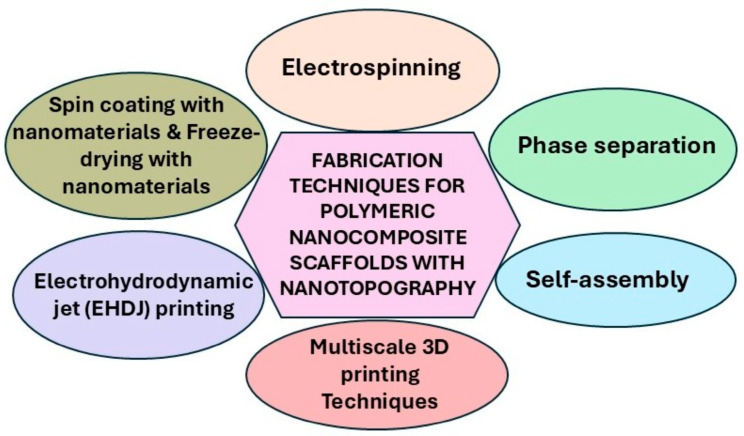
Overview of various fabrication techniques used for producing polymeric nanocomposite scaffolds with nanotopographical features.

Surface topographies play a crucial role in influencing cell behavior and stem cell differentiation. These topographical features can be classified into continuous topographies, such as alternating grooves and ridges, gratings, and parallel fibers; discontinuous topographies, including pillars, posts, cones, and various linear, circular, and dot-shaped patterns; and random topographies, such as nanoroughness [54]. While advanced nanofabrication techniques such as lithography, etching, and self-assembly enable the precise development of these topographies, they often require sophisticated equipment. Alternatively, polymeric scaffolds with controlled sub-micron and nanoscale features can be fabricated using cost-effective top-down and bottom-up approaches, including electrospinning, electrohydrodynamic (EHD) jet printing, phase separation, 3D printing, self-assembly, freeze-drying/lyophilization, and molding/casting techniques (Table 1; Figure 2).

Electrohydrodynamic (EHD) techniques are advanced fabrication and manipulation methods that utilize electric fields, surface charges, or electric currents to manipulate matter―particularly liquids, particles, or jets―for microscale and nanoscale fabrication, patterning, or deposition. Key classes of EHD techniques include electrospinning, EHD jet (EHDJ) printing, and EHD spraying [55]. Among these, EHDJ printing has emerged as a promising 3D printing approach capable of fabricating scaffolds with highly oriented fibers deposited in a layer-by-layer manner, enabling the construction of customized microscale architectures [56]. Jing et al. [57] demonstrated a novel strategy employing EHDJ printing to fabricate poly(ɛ-caprolactone) (PCL)/gliadin composite scaffolds with well-aligned microfibers. By selectively leaching the gliadin phase post-printing, nanoscale pores, and surface irregularities were introduced into the PCL fibers, generating complex fiber nanotopographies. These dual micro- and nanoscale features provided an enhanced 3D cell culture platform, significantly improving cell–scaffold interactions. The resulting scaffolds exhibited a well-defined lattice microstructure with an average pore size of approximately 177 µm. Each side wall was composed of 12 stacked fibers and an average fiber diameter of about 10 µm. In another study, EHDJ 3D printing was utilized for the direct fabrication of PCL/polyvinylpyrrolidone (PVP) composite scaffolds with microscale resolution and a high aspect ratio of ~30. These scaffolds exhibited controllable filament diameters (~10 µm) and demonstrated excellent biocompatibility, supporting increased cell density over time―highlighting their potential for advanced tissue engineering applications [58].

Electrospinning is a versatile and scalable technique for producing ultrathin polymeric fibers with sub-micron and nanoscale dimensions, closely mimicking the extracellular matrix (ECM) architecture for various biomedical applications. This involves an electrohydrodynamic process, where a polymer solution is electrified to generate a charged jet that undergoes elongation and thinning to form fibers [59]. Moreover, the morphology and diameter of electrospun fibers are influenced by processing parameters such as applied voltage, polymer solution flow rate, and the distance between the spinneret tip and the collector. Additionally, other influences include environmental factors, polymer composition, molecular weight, concentration, viscosity, surface tension, dielectric constant, and conductivity affect fiber formation [60]. In conventional electrospinning, a polymer solution is ejected through a single nozzle under high voltage. However, several advanced electrospinning techniques allow for tailored nanofiber morphologies, including co-axial, tri-axial, multi-jet, needleless (or free surface), and melt electrospinning. Co-axial electrospinning enables the fabrication of core–shell fibers by simultaneously spinning two polymers through a dual-layer nozzle, while tri-axial electrospinning involves three polymer layers for complex fiber architectures of core, intermediate, and shell layers. Multi-jet electrospinning utilizes multiple nozzles to produce fibers in high throughput. Needleless electrospinning replaces traditional nozzles with a rotating drum to generate multiple fiber jets for high productivity. Melt electrospinning uses molten polymers instead of solvents, avoiding issues related to solvent toxicity and post-processing. Overall, electrospinning and electrospraying techniques produce nanofibrous biomaterials with diverse topographies, including randomly oriented, aligned, porous, and beaded structures for tissue engineering applications [61,62].

Three-dimensional printing provides precise control over macrostructure (pore size, shape) and microstructure features (surface roughness, texture), influencing cell behavior and ECM deposition [63]. Layer-by-layer (LbL) printing techniques such as digital light processing (DLP)—vat photopolymerization, selective laser sintering (SLS), two-photon polymerization (2PP or TPP), and material jetting and micro-stereolithography (µSL) are commonly employed to fabricate scaffolds with well-defined macro- and microstructures as well as micro/nanopatterning topographical surfaces including smooth, rough, or hierarchical structures [64,65,66]. Phase separation, a thermodynamic process, involves the decomposition of a homogeneous mixture into two or more distinct phases with different compositions. This process can be triggered by alterations in temperature, solvent composition, or chemical interactions, and is widely used to control the shape, size, and spatial distribution of nano- and microscale features of scaffolds. Tuning factors such as polymer concentration, solvent/non-solvent type, and ambient temperature or humidity during processing can yield diverse topographical architectures [67]. Schaub et al. [68] reported an electrospinning approach that leveraged phase separation to precisely tailor the surface topography of individual fibers. By introducing a small amount (<2 wt%) of a non-solvent (dimethyl sulfoxide, DMSO) into a poly(L-lactic acid) (PLLA)/chloroform electrospinning solution, nanoscale depressions (1.88 ± 0.45 µm) were created on the fiber surfaces. These topographical cues significantly influenced the behavior of RAW 264.7 macrophages, altering cell spreading dynamics while maintaining cell adhesion. More recently, Kocourkova et al. [69] demonstrated a novel surface engineering strategy for protein-based biomaterials by applying various phase separation-based techniques to silk fibroin films. This approach enabled the generation of macro-, micro-, and nanostructured surfaces in a hierarchical manner. The engineered fibroin films exhibited long-term stability in physiological conditions and significantly enhanced the adhesion and proliferation of human keratinocytes and fibroblasts, underlining the potential for regenerative medicine.

Self-assembly is a bottom-up fabrication technique that relies on the spontaneous assembly of molecules into organized, well-defined structures through their non-covalent interactions, such as hydrogen bonding, van der Waals forces, electrostatic interactions, and hydrophobic interactions [70,71,72,73]. Self-assembling biomaterials, particularly peptide-based self-assembled structures, form nanofibers, nanotubes, hydrogels, or micelles, driven by the assembly of peptides into beta-sheet, alpha-helix, or random coiled structures, depending on their amino acid sequence and environmental factors like pH and ionic strength. The first reported self-assembling designer peptide, EAK16-II (AEAEAKAKAEAEAKAK), derived from yeast protein zuotin, spontaneously self-assembles into stable β-sheets in aqueous solutions across a broad range of pH and temperature [74]. Xiang et al. [75] demonstrated that the self-assembly behavior of two peptide-functionalized polymeric nanoparticles (peptide1: H_2_N-TTTT-AEAEAEAE-amide; and peptide2: H_2_N-TTTT-AKAKAKAK-amide) with different particle sizes forms “nanoparticle-fibers”, which modulated scaffold porosity, mechanical properties, and biocompatibility with NIH3T3 fibroblasts. Chen et al. [76] engineered a multifunctional fusion protein (rhCR) hydrogel scaffold for wound healing and hemostatic effects by combining recombinant human collagen (rhCol) with the self-assembling peptide RADA16 (RADARADARADARADA) using yeast *Pichia pastoris* expression systems. Similarly, Song et al. [77] developed a peptide nanofiber gel (RGJ) by incorporating bioactive peptides A8SGLP-1 (G) and Jagged-1 (J) into self-assembled peptide RADA16-I (R) in specific ratios, which exhibited stable β-folded structures at room temperature. These RGJs reduced wound and systemic inflammatory responses by promoting the secretion of pro-angiogenic factors such as VEGF and CD31 and accelerated burn wound healing by increased collagen deposition with reduced wound scar formation. In 2019, Yoshimatsu and coworkers demonstrated that self-assembling peptide RADA16-I hydrogels were effective for recurrent laryngeal nerve (RLN) regeneration and thyroarytenoid muscle atrophy [78]. In another instance, PuraMatrix (BD Biosciences, San Jose, CA, USA), a 16-amino acid synthetic peptide (Ac-[RADA]_4_-CONH_2_), which is known to undergo self-assembly into nanofiber hydrogels, forms an interconnected hydrogel network with an average pore size of 5–200 nm and a nanofiber diameter of 10–100 µm by mimicking the ECM [79]. Akiyama et al. [80] demonstrated the in situ tissue engineering by the encapsulation of middle-ear mucosal epithelial cells within PuraMatrix as a nanofiber scaffold for the regeneration of surgically damaged mucosa of the middle-ear in Sprague Dawley (SD) rats through transplantation. Furthermore, MAX8, a designer self-assembling stimuli-responsive peptide (VKVKVKVKV^D^PPTKVEVKVKV-NH2, wherein ^D^P is D-proline) undergoes gelation under physiological conditions by self-assembling into 3.2 nm diameter β-hairpin nanofibers [81,82]. Worthington et al. [83] used peptide-based shear-thinning MAX8 hydrogel tagged with the RGDS (Arg-Gly-Asp-Ser) sequence to create a synthetic ECM scaffold for 3D cell culture, demonstrating the potential for high-throughput drug screening (HTS) applications for medulloblastoma, a pediatric brain cancer. Moreover, due to the folding and self-assembling kinetics of MAX8, gel-cell constructs were developed for the delivery of encapsulated cells to target biological sites for tissue regeneration [81].

Another important fabricating technique to manipulate polymeric nanocomposite scaffold topographies is through solvent casting and particulate leaching (SCPL), spin coating with nanomaterial techniques [84,85]. In these techniques, the polymeric solution in a solvent is cast into a mold with the incorporation of particulate leaching agents, which are subsequently leached out by dissolution to create a porous architecture. These techniques enable the fabrication of scaffolds with macro/microporous structures. Freeze-drying (lyophilization) is another effective approach, where a polymer solution is frozen with nanomaterials and then sublimated under vacuum, yielding porous networks [86]. The surface texture and roughness of scaffolds can be further modulated by controlling solvent evaporation rates and cooling conditions, allowing for tailored biomaterial properties suited for specific biomedical applications.

**Table 1:** Fabrication techniques for polymeric nanocomposite scaffolds with nanotopographical features and their advantages.

Fabrication Technique	Nanotopographical Dimension	Advantages	References
Electrohydrodynamic jet (EHDJ) printing	~100 nm–1 µm	Precise deposition of biomaterials and nanoparticles, tunable feature sizes	[55]
Electrospinning	Nanofibers: 50–500 diameter	Mimics native ECM architecture; high surface area-to-volume ratio	[59]
Phase separation	Nanopores: 50–200 nm	Produces porous, interconnected structures	[67]
Self-assembly	Nanodomains: 10–100 nm	Mimics natural ECM at the nanoscale; tailorable surface chemistry	[70,74]
3D printing (two-photon polymerization)	~100 nm	Fabricate complex 3D structures, with high spatial control with porosity and architecture	[63]
Layer-by-layer (LbL) assembly	Nanolayers: ~1–10 nm/layer	Molecular-level control, functional multilayer coatings, versatile biomolecule incorporation	[66]
Spin coating with nanomaterials	Nanoroughness: 10–100 nm	Tunable surface roughness	[85]
Freeze-drying with nanomaterials	Nanoscale structures	High porosity, nanoroughness	[86]

## 5. Applications of Nanotopography of Polymeric Composite Scaffolds in Tissue Engineering and Regenerative Medicine

The surface nanotopography of a substrate plays a pivotal role in regulating stem cell fate by reorganizing the cytoskeleton and assembling intracellular focal adhesion proteins, thereby modulating mechanotransduction pathways [87]. These topographical features significantly influence cell morphology, alignment, and orientation, ultimately affecting lineage-specific differentiation. Additionally, the mechanical tension generated by specific surface topographies initiates integrin-mediated intracellular signaling cascades, which influence nuclear organization and drive centromere repositioning through nuclear deformation [88]. This sequence of events alters the expression of genes associated with proliferation, differentiation, and cellular phenotype, thereby shaping cell function in tissue development and regeneration.

The architectural and structural features of scaffolds―such as geometrical motifs, contours, and surface roughness―strongly modulate cellular responses under both physiological and pathological conditions [89]. While numerous studies have explored the impact of nanoscale substrate topography on cell proliferation and differentiation, the underlying molecular mechanisms remain incompletely understood. Incorporating nanoparticles into polymeric scaffolds introduces nanoscale surface roughness, which enhances mechanical strength while providing bioactive cues that regulate cell interactions [49]. Notably, nanoscale roughness in the range of 10–200 nm has been shown to enhance cell adhesion and promote osteogenic differentiation of human mesenchymal stem cells (hMSCs) by mimicking the extracellular matrix (ECM) topography [39]. Furthermore, aligned nanotopographies can direct cell migration, a critical factor in wound healing and tissue regeneration [90]. An in-depth understanding of these topographical effects will enable the development of advanced polymeric composite scaffolds tailored for specific applications in tissue engineering and regenerative medicine (Table 2).

### 5.1. Organic Nanomaterial-Modified Topographies

Organic nanoparticles derived from natural and synthetic polymers, such as chitosan, dextran, alginate, collagen, gelatin, polydopamine, cellulose, and polypyrrole, can modify the topography of polymeric composite scaffold, mimicking the ECM architecture to regulate cellular interactions, bioactivity, and cell behavior (Table 2). Among these, chitosan, a deacetylated form of chitin, is the second most abundant natural polysaccharide on Earth after cellulose. It is composed of a linear, semicrystalline polysaccharide structure with randomly distributed β-(1 → 4) linkages of N-acetyl-glucosamine and glucosamine, making it a non-toxic and biodegradable polymer widely used in fabricating natural composite biomaterials [91]. Chitosan-organic composites can be processed into hydrogels, sponges, membranes, or nanofibers for various biomedical applications. Bakhshandeh and colleagues developed a polymeric artificial cornea using dextran/chitosan (DEX-CS) nanoparticles (d = 212 nm) encapsulated with human amniotic membrane extract (hAME), which inhibits inflammation, angiogenesis, and scarring, which induces epithelial formation and was physically decorated onto a poly(Ɛ-caprolactone) (PCL) nanofibrous scaffold thermally connected to a synthetic biocompatible poly(vinyl alcohol) (PVA)-based hydrogel disk. The resulting biocompatible and transparent biomaterial holds promise for corneal transplantation, exhibiting nanotopographical architecture suitable for human umbilical vein endothelial cells (HUVECs) [92]. Berberine, a plant alkaloid known to promote axonal growth and regeneration in damaged peripheral and central nerve systems (CNSs), has been successfully encapsulated within chitosan nanoparticles (BerNCh) through polyelectrolyte interaction and ionic gelation. Further encapsulation of BerNCh into a hybrid alginate–chitosan (Alg-Ch) hydrogel (Alg-Ch/BerNCh) by polyelectrolyte complex gelation facilitated spinal cord regeneration by promoting endometrial stem cell adhesion and restricting secondary damage [93]. Similarly, spherical ZnO and chitosan nanoparticles (CS NPs) (~20 nm and ~11.98 nm, respectively), synthesized via sol–gel and ionotropic gelation methods, were incorporated into chitosan scaffolds using a freeze-drying technique. The resulting porous morphology (100–450 µm) demonstrated antibacterial activity and biocompatibility, making it suitable for tissue engineering applications [94].

Extensive bone damage significantly diminishes vascularization and nutrient supply to the injury site, thereby reducing the inherent capacity for self-generation and treatment. Biomaterial scaffolds can deliver nanomedicines to stimulate bone regeneration, particularly by leveraging the role of microRNAs (miRNAs) in osteogenesis [95]. In particular, miR-133a, miR-16, and miR-138 have been shown to enhance osteogenic differentiation [96,97,98], while miR-126 and miR-210 regulate vascular integrity and developmental angiogenesis, and vascular endothelial growth factor (VEGF)-driven cell migration, respectively, by stimulating the expression of VEGF or hypoxia-inducible factors (HIFs) [99,100]. Recently, miR-26a gene therapy has demonstrated osteogenic and angiogenic potential in inducing osteodifferentiation of MSCs and promoting bone formation [101]. However, miRNA stability remains a key challenge in various therapies. To address this, Sadowska et al. [102] developed a nanoparticle-based delivery system for miR-26a, achieving in vivo bone regeneration. Collagen, one of the most abundant natural polymers, forms 3D matrices made up of nanofibrillar structures. Incorporation of miR-26a complexed cell-penetrating peptide (RALA) nanoparticles into collagen–nanohydroxyapatite (coll-nHAp) scaffolds significantly enhanced the production of VEGF, ALP activity, osteogenic differentiation, mineralization, and angiogenic factor release for highly mineralized and vascularized bone tissue engineering for hMSCs through osteogenic–angiogenic coupling in the repair of critical-sized bone defects.

**Figure 3:**
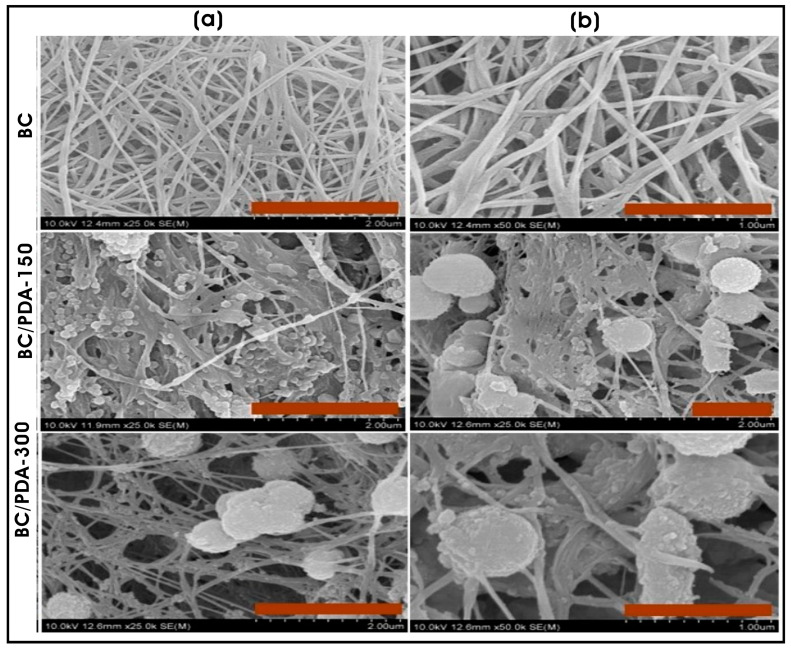
(**a**,**b**) FE-SEM images of BC and BC/PDA hydrogels at different magnifications. Scale bars: (**a**) 2 µm, (**b**) 1 µm. (**a**,**b**) Reproduced with permission from [104].

Electrospun PCL–gelatin nanofibrous scaffolds combined with collagen nanofibers or nanoparticles were fabricated using dual-pump electrospinning to evaluate their effects on the cell adhesion and behaviors of normal human epidermal keratinocytes (NHEK). The results indicated that scaffolds with surface topography created by collagen nanoparticles exhibited significant improvement in cell viability, adhesion, and spreading of cells compared to collagen nanofiber-based scaffolds [103]. Similarly, a bacterial cellulose hydrogel scaffold functionalized with polydopamine (PDA) micro/nanospheres demonstrated enhanced NIH/3T3 fibroblast adhesion and proliferation, with larger PDA spheres (0.65 ± 0.14 µm) providing a more favorable microenvironment than smaller PDA spheres (0.25 ± 0.18 µm), which suggested that topography of BC hydrogel regulates the proliferation and survival of embryonic mouse fibroblasts [104] (Figure 3a,b). Additionally, Narayanan and coworkers leveraged the topography of filamentous fungal morphology composed of chitin-glucan nano-/microfibrous spheres with the surface coating of collagen type I (C-FNS; ~363 ± 61 nm) were employed as scaffolds for fibroblast spheroid formation, highlighting their potential in tissue engineering and regenerative medicine [105]. Earlier, they utilized the filamentous fungal scaffold of *Aspergillus* sp., mimicking ECM architecture, as a 3D biomaterial for the culture of human keratinocytes for skin tissue engineering applications [106].

Scaffold porosity significantly influences osteoblast proliferation and differentiation. Furthermore, the surface chemistry and topography of scaffold matrices influence in vivo bone formation, osseointegration, and bone binding [107]. Pudelko et al. [108] fabricated porous zirconia (ZrO_2_) scaffolds using a foam replication method and surface-functionalized with a calcium phosphate (CaP) layer containing gentamicin-loaded PLGA nanoparticles. These scaffolds, mimicking spongy bone microarchitecture, exhibited cytocompatibility with osteoblast-like MG-63 cells while providing antimicrobial properties against bone implant-related infections. For neural tissue engineering, scaffolds must exhibit aligned fibrous structures with electrical conductivity and antioxidant properties. Electrospinning combined with electrospraying enables the fabrication of biomimetic scaffolds with aligned fibers with electrical conductivity. Tang et al. [109] developed highly aligned PCL microfibrous neural scaffolds with co-sprayed collagen and electroconductive and antioxidant difunctional polypyrrole nanoparticles (PPy NPs; d = ~70 nm), which facilitated topographical guidance, fiber conductivity for electric signals and good bioactivity for neurogenesis. The aligned fibrous topography promoted the elongation of PC12 along the direction of fibers of aligned PCL scaffolds (diameter of aligned fiber: 5.17 ± 0.3 µm) inducing neurogenesis for neural tissue engineering. Similarly, Wu et al. [110] developed 3D-printed PPy NP-embedded methacrylate anhydride (MA)-modified hyaluronic acid (HA) (HAMA)–collagen hybrid hydrogel, which transmitted the intercellular and external electrical signals and promoted neuronal differentiation of BMSCs through PI3K/Akt and MAPK signaling pathways for spinal cord injury (SCI) repair.

**Figure 4:**
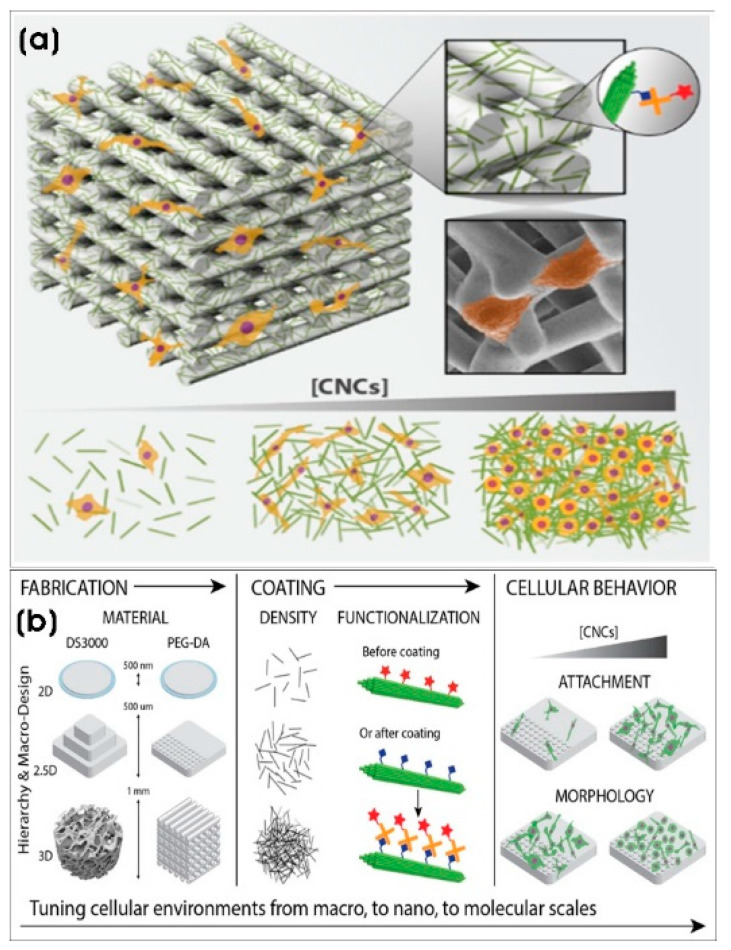
(**a**) Tuning the nanotopography and chemical functionality of 3D-printed DS3000 and poly(ethylene glycol)diacrylate (PEG-DA) scaffolds via cellulose nanocrystal (CNC) coatings. (**b**) The fabrication of cellular microenvironments with tunable nanotopography and functionality. Scaffolds of varying complexity, from 2D thin films to 2.5D and 3D structures, were fabricated using spin coating (followed by flood exposure to UV light) and one- or two-photon 3D printing techniques. The topography of these scaffolds can be precisely tuned through layer-by-layer (LbL) deposition of CNC coatings, with surface coverage ranging from sparse to dense. The scaffold surface can be functionalized with a specific chemical moiety either by direct grafting on the CNC surface or by coating with a molecule that targets the functionality encoded on the CNCs. The density of the CNC coating on the 3D-printed scaffolds can be finely adjusted, enabling precise control over cell adhesion and phenotype. Adapted with permission from [114]. Copyright 2021, American Chemical Society.

Drug-loaded nanoparticles that functionalized composite scaffolds can be used in regenerative medicine. For instance, carvacrol (CA), an anti-inflammatory and anticancer drug, was loaded into lipid nanoparticles (CA-LNPs; ~129 nm) and incorporated into 3D-printed SiO_2_-doped β-tricalcium phosphate (β-TCP) scaffolds, significantly increasing osteoblast proliferation by 2-fold while reducing osteosarcoma cell viability by 3-fold [111]. Similarly, Bose and colleagues demonstrated that increasing the external surface area by 40% and topography of binder jet-based 3D-printed porous β-tricalcium phosphate (β-TCP) cylindrical scaffolds resulted in a two-fold increase in osteoblast proliferation in vitro, suggesting site-specific bone defect repair and other bone grafting applications [112]. Cellulose nanostructures are relatively abundant homopolysaccharide biopolymers composed of β-glucose molecules offering high mechanical strength and biodegradability. Nanocellulose-based composites provide tunable surface topographies and chemical functionalities [113]. Babi et al. [114] demonstrated that dip-coating 3D-printed DS3000 and poly(ethylene glycol)diacrylate (PEG-DA) scaffolds with cellulose nanocrystals (CNCs) (length × width; 100–200 nm × 5–20 nm) with varying densities of CNC altered surface nanoroughness (RMS and surface chemistry of phenotypic morphology of adhered prostate cancer cells) provides nanostructured topography. Moreover, the chemical functionalization of these scaffolds with biotin-CNC allows conjugation with streptavidin-conjugated molecules to recreate real cellular environments in vitro to create artificial tissues. This microenvironment with the cells, which is a well-organized framework or scaffold that constitutes living tissue should be replicated or fabricated in vitro as scaffolds with tunable properties for adhesion, proliferation, functions, and other cell behavior [115] (Figure 4).

### 5.2. Inorganic Nanomaterial-Modified Topographies

Inorganic nanoparticles, including hydroxyapatite, metals, metal oxides, natural clay minerals, bioactive glass, and ceramics are frequently incorporated into polymeric composite solutions to fabricate nanoscale roughness with conductive nanostructures or hierarchical microporous and nanoporous surfaces (Table 2). Such engineered biomaterials exhibit enhanced bioactivity, osteoinductivity, and osteoconductivity, making them highly suitable for bone defect degeneration and also for various other tissue engineering applications [116].

Ceramic nanoparticles play a crucial role in bone regeneration. For instance, bredigite (Bre) nanoparticles, when doped with strontium (Sr) (Bre-Sr), yield a novel nanocomposite scaffold (PLA/PCL/Bre-Sr) fabricated using poly(Ɛ-caprolactone) (PCL) and poly (lactic acid) (PLA) through 3D printing. These scaffolds with incorporated Bre-Sr ceramic nanoparticles of less than 200 nm in size exhibited an average pore size of approximately 400 µm. The resulting topography of these scaffolds promotes excellent bone tissue regeneration by enhancing human osteoblast adhesion and proliferation [11]. The scaffold topography critically regulates cell adhesion, proliferation, and functions. Biomaterials with multifunctional properties―such as osteogenesis enhancement, osteoclastogenesis inhibition, and antibacterial capabilities―are necessary for treating osteoporotic bone defects [117]. Additionally, scaffold porosity is crucial in determining cell migration and nutrient diffusion to the cells within 3D scaffold structures. Moreover, surface topography, mechanical stiffness, surface chemistry, and biological components within 3D microenvironments significantly influence cellular phenotypes and functional behavior [118]. Various cell types, including macrophages, actively sense micro and nanoscale topographical features, influencing their adhesion and morphology. For example, murine bone marrow-derived macrophages cultured on nanostructured SiO_2_ films exhibit increased membrane protrusions, while microstructured surfaces promote start-shaped morphologies [119]. Incorporating uncoated and Al_2_O_3_- and SiO_2_-coated barium titanate nanoparticles (BTNPs) (with an average size of 50–80 nm and a round morphology, while some are oval and angular-shaped nanoparticles) into poly-L/D-lactide copolymer (PLDLA) scaffolds using the breath figure method results in a porous honeycomb-like structure. These 3D PLDLA/BTNP composite scaffolds demonstrated an average pore size of 16.2 µm and exhibited excellent biocompatibility. Furthermore, ovine bone marrow stromal stem cells adhering to these scaffolds exhibited a spherical morphology, suggesting favorable cytocompatibility [120]. Further innovations include the in situ decoration of silicon dioxide (SiO_2_) nanoparticles on the surface of graphene oxide (GO) (GO@SiO_2_) nanosheets. This modification increased the interlayer distance of GO nanosheets from 0.799 nm to 0.894 nm, enhancing their interaction with poly(L-Lactic acid) (PLLA) for the fabrication of bone scaffold. PG10 scaffolds developed from this composite exhibited excellent cytocompatibility and promoted the viability of human MG-63 osteoblastic cells for bone tissue engineering [121].

Magnesium (Mg^2+^) ions, known for their anti-inflammatory and osteogenic potential, further enhance scaffold functionality. Intriguingly, silver ion (Ag^+^) adsorption induces morphological transformation in cube-shaped MgO NPs, forming lamella-structured MgO-xAg nanocomposites (NCs). Among these, MgO-1Ag NCs showed enhanced cell viability of human osteoblastic SaOS-2 cells and significantly stimulated their proliferation and differentiation. Gene expression studies revealed the upregulation of osteogenic markers such as alkaline phosphatase (ALP), collagen (COL), and osteoprotegerin (OPG), all critical to the differentiation of SaOS-2 cells [122]. Similarly, Wu et al. [117] demonstrated that lamella-shaped MgO-xCu (x = 0.1, 1.0, and 10) NCs―composed of amorphous Cu(OH)_2_, crystalline Mg(OH)_2_, and minor MgO―modulate osteoblast and osteoclast responses in a cupric cation (Cu^2+^)-concentration-dependent manner. MgO nanoparticles (average size: 34.2 nm) loaded with Cu^2+^ transformed from a plate-like shape into MgO-xCu nano-lamellae, with MgO-10Cu NC (101.2 × 9.6 nm; length × thickness) exhibiting a stronger stimulatory effect on osteoblast proliferation and differentiation while effectively inhibiting osteoclast formation.

Hydroxyapatite nanoparticles (nHAp), chemically similar to natural bone composition, possess osteoinductive potential by adsorbing growth factors and facilitating osteoblast differentiation and mineralization [123]. In bone tissue engineering, mimicking the ECM of a bone environment requires a combination of both organic and inorganic materials. The inorganic/polymeric hybrid nanocomposite composed of hydroxyapatite/polycaprolactone nanoparticles (HAp/PCL NPs) augmented superior osteogenicity, particularly with spherical nanoparticles (176.24 ± 40.75 nm). The HAp nanoparticles within the composite had a rod-like morphology with a length and width of 92.18 ± 18.21 nm × 30.3 ± 4.7 nm, respectively. These HAp/PCL NPs as a nanoplatform exhibited good cytocompatibility, proliferation, and osteodifferentiation of MSCs. There was an elevated gene expression of early osteogenic markers (Runx-2 and osteopontin) and a late osteogenic marker (bone sialoprotein), which confirms the enhanced osteogenic potential of hybrid bioactive HAp/PCL NPs nanoplatform. The incorporation of HAp nanoparticles into the porous PCL scaffolds provided a favorable surface topography and chemical constitution, creating a favorable environment for osteogenesis compared to unmodified PCL scaffolds [124]. Interestingly, the nanotopography surface of PLA electrospun microfiber matrix, incorporating streptomycin (STR)-loaded hydroxyapatite nanoparticles (HAp nanorods with a diameter of 20–50 nm and a length of 50–150 nm) along with aggregated HAp nodules, provides scaffolds for tissue engineering with antibacterial and antitumor properties [125].

The nanostructured surfaces can be fabricated by various techniques, and the incorporation of surface nanostructures alters surface roughness, playing a key role in modulating cell behavior and function [126]. The electrospinning technique has been used to prepare nanofibrous membranes that structurally and functionally mimic the natural bone matrix, facilitating adhesion, proliferation, and osteogenic differentiation of bone cells. Whitlockite is a naturally occurring phosphate mineral composed of calcium magnesium phosphate with the chemical formula Ca_18_Mg_2_(PO_4_)_12_[PO_3_(OH)]_2_. It is found in biological systems, particularly in bone and dental enamel [127,128]. Zhang et al. [129] developed a biofunctionalized poly(ɛ-caprolactone) (PCL) nanofibrous membrane loaded with tantalum (Ta)/whitlockite (WH) nanoparticles (PCL/Ta/WH) using electrospinning technology, which promotes bone defect repair through neurovascular processes. The release of Mg^2+^ from WH effectively increases the local nerve and vascular density, while its combination with Ta NPs creates a nerve–vascular microenvironment that enhances the osteoinduction process, aiding in the repair of complex critical bone defects. Steroid-associated osteonecrosis (SAON) is a chronic disease that leads to the destruction and collapse of bone near weight-bearing joints, leading to loss of hip and knee function. Elemental zinc plays a crucial role in enhancing bone regeneration and improving the immunophysiological cellular environment while also exhibiting antibacterial and antibiofilm properties. Inorganic clay minerals are interesting nanofillers incorporated in polymeric nanocomposites, which enhance mechanical properties, surface topography, roughness, and texture [130]. Natural clay mineral nanocomposites have been incorporated into biodegradable polymer composites such as PLA [131], starch [132], chitosan [133], and PHAs [134]. Interestingly, willemite (W), a zinc-based silicate mineral (Zn_2_SiO_4_), and these biomaterials have been shown to influence gene expression, protein synthesis of osteoblasts, and calcification [135]. Recently, Bardeei et al. [136] prepared a PCL/nanoparticulate willemite (npW) (PCL/npW) composite scaffold using 3D printing technology, which enhanced the cell viability and biocompatibility. Importantly, compared with neat PCL scaffolds, the surface modulation and topography of PCL/npW promoted osteoblast differentiation and hydroxyapatite accumulation, facilitating new bone formation and regeneration. This makes it a promising bone substitute for early SAON treatment and other osteonecrosis-related defects.

Extensive research has been conducted on the interaction of metal/metal oxide nanoparticles with biological tissues. These metal/metal oxide nanoparticles, in conjunction with nanotopography, facilitate electrostatic and van der Waals interactions that modulate cell behavior. Various metal and metal oxide nanoparticles, including silver (Ag), gold (Au), iron oxide (Fe_2_O_3_), titania (TiO_2_), zinc oxide (ZnO), and ceria (CeO_2_), have demonstrated significant tissue regeneration potential [137,138,139,140]. Various other nanoparticles also influence tissue regeneration through their unique topographical features, piezoelectric properties, and electrical conductivity [141]. For instance, a nanocomposite conduit composed of spherical ZnO NPs (30 nm in diameter) and chitosan (CZON) significantly improved the functional recovery of a dissected left sciatic nerve defect in rats. The nanocomposite successfully bridged a 10 mm sciatic nerve defect with enhanced motor and sensory regeneration and reinnervation following the repair of a peripheral nerve by an increased number of axons and their diameter compared to the repair by chitosan-only conduit without nanoparticles [142]. Additionally, self-powered biomimetic piezoelectric nanogenerators made of ZnO NPs/PCL scaffolds were fabricated using 3D injectable multilayer biofabrication. The incorporation of spherical ZnO NPs (30–80 nm) within and on the surface of the scaffold enhanced neuronal growth, the differentiation of PC12 cells, and the proliferation of Schwann cells compared to PCL-only scaffolds. Moreover, the electricity generated by the ZnO-loaded scaffold stimulated the protuberance extension of neurons and increased cellular adhesion on the material surface. These ZnO NPs/PCL scaffolds also increased the number and diameter of myelinated axons as well as the thickness of myelin sheaths [143]. Furthermore, PCL composite nanofibers surface-modified with incorporated ZnO NPs and dual-coated with polydopamine (PDA) and QK peptides (PCL@Z/P/QK) demonstrated remarkable hydrophilicity, biocompatibility, antibacterial properties, and osteo-angiogenesis potential. These properties make these scaffolds a promising candidate for orthopedic implants and bone defect repair [144]. The continued exploration of nanoscale structures in scaffold design as topographical entities presents a potential to revolutionize tissue engineering and regenerative medicine by improving cellular interactions, facilitating guiding differentiation, and supporting targeted therapeutic interventions.

Contrary to the reported toxicity of various metallic nanoparticles, cerium oxide or ceria nanoparticles (nanoceria; CeO_2_ NPs) exhibit superior neuroprotective properties, making them promising candidates for therapeutic applications [145]. Nanoceria (5–80 nm) has been shown to reduce the gene expression of glutathione synthetase (GSS) and glutathione peroxidase 1 (GPx1), thereby mitigating apoptosis and promoting neurite outgrowth with an increase in the neurite length from 55 to 85 µm. Moreover, nanoceria possesses potent free radical-scavenging abilities and also enhances the differentiation of nerve cells, contributing to neural regeneration [146]. The development of functionally advanced scaffolds capable of actively interacting with stem cells holds great potential for overcoming the limitations of current stem cell-based therapies. Nanoceria provides nanoscale redox reaction sites, functioning as antioxidant enzymes that facilitate angiogenesis and support the formation of complex neural networks [147]. The incorporation of nanoceria and graphene oxide (GO) sheets into 3D composite scaffolds with tunable mechanical properties, porous geometry, and electrical conductivity has been shown to promote antioxidant activity and neuroprotection. These properties facilitate the differentiation of neural stem cells into neuronal, astroglial, and oligodendroglial lineage cells, contributing to the restoration of the injured central nervous system (CNS) [148]. Electrospun, highly aligned composite gelatin/nanoceria nanofiber scaffolds fabricated using electrospun nanoceria (<5 nm) exhibited self-generative antioxidant properties with strong free radical-scavenging abilities, promoting neurite sprouting for nerve tissue engineering and regenerative medicine. A topographic anisotropy-dependent enhancement of axonal outgrowth was observed in both the presence and absence of nanoceria. Notably, there was an increase in the length of axons on aligned nanoceria nanofibers (21.5 ± 1.7 µm) compared to the randomly aligned nanoceria scaffolds (16.2 ± 1.1 µm). Both these scaffolds showed longer neurites than gelatin substrates without nanoceria, highlighting the positive effect of nanoceria in neuronal development [149].

**Figure 5:**
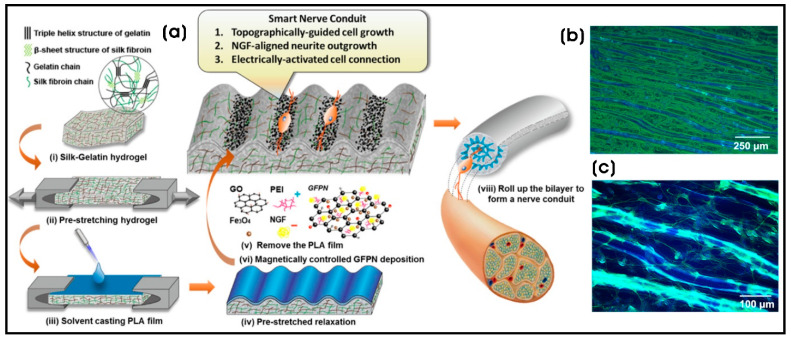
(**a**) A schematic illustration of the experimental process: (i) the synthesis of SG hydrogels; (ii) the hydrogels were pre-stretched to 50% after swelling; (iii) 20 μL of PLA solution was applied onto the pre-stretched hydrogel; (iv) the pre-stain was released, and one-dimensional ordered corrugation patterns were formed; (v) PLA film was retrieved directly; (vi) the rGO–Fe_3_O_4_–PEI–NGF (GFPN) complexes were magnetically and topographically induced to deposit in the grooves of the corrugation patterns, followed by PC12 cell adhesion; (vii) electrical stimulation was applied to the cells; (viii) the bilayer was rolled up to form a nerve conduit. (**b**,**c**) Fluorescent images demonstrating cellular orientation at day 21 after electrical pulse (EP) stimulation on GFPN-coated SG hydrogels. Adapted with permission from [154]. Copyright 2020, American Chemical Society.

Incorporating nanoscale structures into artificial scaffolds plays a pivotal role in modulating cell phenotypes. Microporous composite scaffolds embedded with nanostructures or nanoparticles that introduce nanotopographies significantly influence the adhesion, proliferation, and functionality of various cell types, making them highly relevant for tissue regeneration and regenerative medicine [150]. Regenerative medicine encompasses cell and gene therapies, including strategies for cancer treatment. The elimination of residual cancer cells in post-surgical or postoperative scenarios is critical, not only to prevent recurrence but also to facilitate the generation of healthy tissue [151]. A promising approach involves Fe_3_O_4_/gelatin (Gel/FeNP) composite scaffolds with free ice microparticle-controlled pore structures. These scaffolds are fabricated by chemically modifying gelatin with folic acid (FA) and hybridizing it with citrate-modified Fe_3_O_4_ nanoparticles (Fe_3_O_4_-citrate NPs) exhibiting a flower-like morphology with an average size of 29.6 ± 3.9 nm. This composite scaffold selectively captures breast cancer cells using FA and kills them through magnetic hyperthermia upon exposure to alternating magnetic field (AMF) irradiation. Furthermore, the 3D FA-Gel/FeNP composite scaffold facilitates the growth and adipogenic differentiation of human bone marrow-derived MSCs (hBM-MSCs), offering potential applications in regenerating breast tissue defects in the post-surgical treatment of breast cancer [152]. Similarly, the poly-L-lactic acid (PLLA) fibrous scaffolds grafted with superparamagnetic iron oxide nanoparticles (SPIONs) demonstrated enhanced neurite outgrowth when stimulated with an alternating magnetic field at 1.48 T. SPIONs were considered aggregated if their cluster area exceeded 0.05 µm^2^. Scanning electron microscopy (SEM) analysis of the composite fiber revealed an average of 6 ± 4 aggregates per 100 µm^2^, with an average aggregate size of 0.52 ± 0.54 µm^2^ [153]. In another instance, a facile, lithography-free micropatterning technique was employed to develop a smart protein hydrogel-based scaffold that facilitated topographical, electrical, and chemical cues for neural regulation. The silk–gelatin (SG)/polylactic acid (PLA) bilayer nanocomposite hydrogel featured SG pores of ~10 µm, forming 3D-ordered, well-defined SG corrugated micropatterns (λ ≈ 30 µm) through stress-induced topography. Magnetically and topographically guided deposition of nerve growth factor (NGF)-incorporated IONPs–graphene nanoparticles (GFPN), modified with polyethyleneimine (PEI), facilitated controlled one-dimensional neuronal adhesion, differentiation, and neurite orientation in response to the pattern guidance along the groove regions of SG. Electrical stimulation of PC12 cells resulted in a 1.5-fold increase in neurite elongation, demonstrating its potential in the development of next-generation nerve conduits [154] (Figure 5). Previous studies have also shown the effectiveness of collagen film topographies decorated with oriented spherical PEG-capped paramagnetic IONPs (PEG@IONPs; 80 nm). These films, arranged in long-range aligned micropatterns along magnetic field lines, exhibited biocompatibility when cultured with mouse fibroblast cells (NIH 3T3), indicating their potential in tissue engineering and regenerative medicine, particularly in applications requiring external electrical stimulation [155]. Due to their metallic properties and nanotopography, IONPs serve as an effective platform for stem cell differentiation [156]. The fabrication of 3D alginate/magnetic short nanofiber (M.SNF) composite hydrogels—composed of alginate hydrogel and M.SNF (222 ± 64 nm) synthesized using wet electrospinning of gelatin nanofibers incorporated with spherical SPIONs (10 ± 2 nm) and fragmented using probe sonication—demonstrated enhanced neuronal differentiation. Encapsulated olfactory ecto-mesenchymal stem cells (OE-MSCs) exhibited increased expression of Nestin, β3-tubulin, and GFAP, indicating differentiation into neuron-like and glial cells [157].

**Figure 6:**
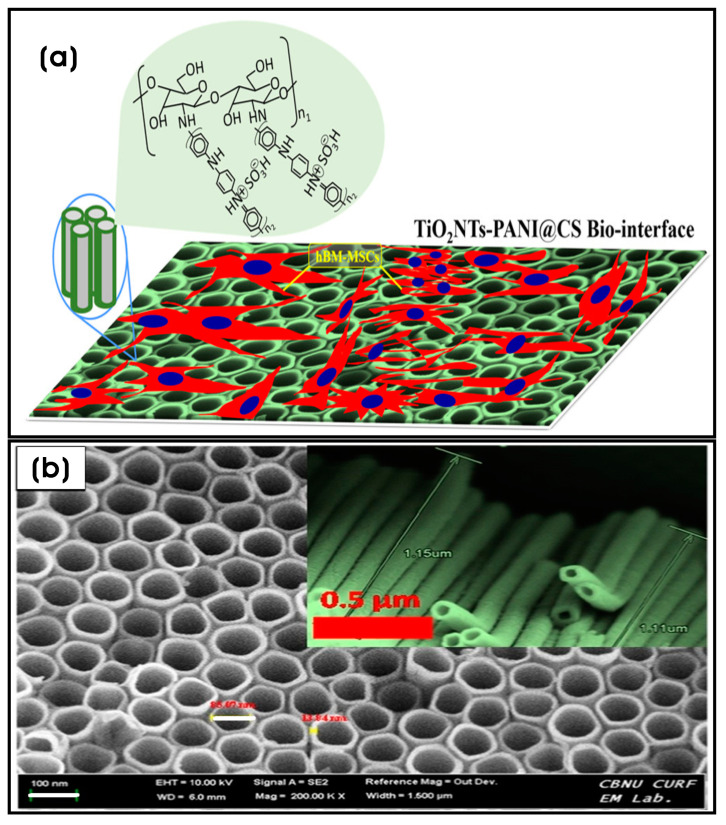
(**a**) The chitosan-crosslinked polyaniline patterning on TiO_2_ nanotubes (TiO_2_NTs-PANI@CS) facilitates the differentiation of human bone marrow mesenchymal stem cells (hBM-MSCs) into osteoblasts. (**b**) FE-SEM images of TiO_2_ nanotubes prepared at 20 V. The inset in (**b**) illustrates the uniform cylindrical morphology of the TiO_2_NT array, with consistent size, length, and diameter. Copyright 2023, MDPI Publisher. (**a**,**b**) Adapted with permission from [161]. Copyright 2021, American Chemical Society.

Titania or titanium dioxide (TiO_2_) coating on various implants confers improved biocompatibility and anti-corrosiveness [158]. Additionally, nanoscale topographies influence cell proliferation and differentiation by triggering molecular mechanisms that sense and adapt to surface properties by activating specific intracellular signals that regulate cell survival and behavior. For example, cluster-assembled nanostructured TiO_2_ (ns-TiO_2_) films obtained by supersonic cluster beam deposition (SCBD), exhibit roughness-dependent neuritogenesis in PC12 cells. Films with 20.2 ± 0.5 nm root mean square (rms) roughness (50 nm thickness) and 29.1 ± 1.0 nm rms roughness (200 nm thickness) triggered neurite outgrowth in PC12 cells without nerve growth factor (NGF) stimulation, mediated by the activation of nitric oxide synthase (NOS) and the phospho-extracellular signal-regulated kinase ½ (pERK1/2) signaling pathway. This effect is more pronounced than on poly-L-lysine (PLL)-coated glass coverslip surfaces or flat TiO_2_ films deposited on glass slides via electron beam evaporation [159]. Titanium-based biomaterials are widely used in hard tissue engineering and implants due to their biocompatibility and osteoregenerative ability [160]. However, Ti-based substrates often fail to interact effectively with surrounding cells, sometimes leading to biomaterial dislocation from the implant site. An engineered osteoconductive and osteoinductive micro-/nanostructured biomaterial composed of chitosan-crosslinked polyaniline (PANI) nanonets coated on titanium nanotubes (TiO_2_NTs) (TiO_2_NTs-PANI@CS) mimics the ECM of bone tissue. This biomaterial promotes hydroxyapatite nanoparticle (nHAp) nucleation and exhibits excellent biocompatibility with human bone marrow-derived mesenchymal stem cells (hBM-MSCs), inducing their proliferation and osteoblast differentiation through the upregulation of osteogenesis-related genes, including collagen-I, osteopontin (OPN), osteocalcin (OCN), and runt-related transcription factor 2 (RUNX2). The cylindrical TiO_2_ NT array (tube wall thickness × length × inner diameter; 13.84 × 1.15 × 85.07 nm), synthesized via electrochemical oxidation at the applied voltage of 20 V at a current of 0.03 A, provides a large surface-to-volume ratio, contributing to a mechanically robust, porous structure with a regular surface topography. This design is well suited to adapt to the mechanical stress exerted by cells on scaffolds and enhances cell–substrate interactions, including proliferation, infiltration, and migration. In addition, the rapid protein adsorption and the deposition of noodle-shaped calcium and phosphate or hydroxyapatite on the scaffold surface confirm the bioactivity and biomineralization capacities of these substrates [161] (Figure 6a,b).

YeAlthough silver nanoparticles (AgNPs) are highly cytotoxic to human cells, they possess high electrical conductivity, making them promising candidates for tissue and nerve regeneration applications. The cultivation of human neuroblastoma cells on AgNP-coated glass at low (10 AgNPs/µm^2^) and high (50 AgNPs/µm^2^) densities resulted in neurite outgrowth enhancements of 2.8-fold and 1.4-fold, respectively, compared to control glass and AuNP-coated glass substrates. These effects are attributed to the nanotopographical modifications induced by the physical characteristics of AgNPs, which provide anchoring sites for neurite extension [162]. Additionally, AgNPs (110 ± 40 nm) at a density of 14 AgNPs/µm^2^ on the glass surface favored the anchoring sites and growth of highly straightened neurites on AgNPs in the elongation phase, whereas cells cultured on control glass substrates developed curved branches. This demonstrated AgNPs as regenerative materials, leveraging nanotopography to promote neuronal growth. However, despite their nanotopographic effects, AuNPs (d =100 ± 40 nm) and ZnONPs (d = 115 ± 45 nm) did not significantly enhance material-driven neurite elongation [163]. The differentiation of neuroblastoma cells into neurites is mediated by the activation of ERK1/2 and AKT pathways via phosphorylation, accompanied by the downregulation of phosphatase expression and increased intracellular reactive oxygen species (ROS) [164]. Furthermore, an electrically conductive implant composed of PEG-based hydrogel with 200 µm ridge-patterned surfaces containing silver nanowires on a flexible polyethylene terephthalate (PET) film was fabricated using PDMS molds. This system significantly enhanced nerve stem cell (NSC)-derived neuronal differentiation and neurite guidance under electrical stimulation, as confirmed by immunostaining with fluorescently labeled antibodies for Tuj1-positive neuronal cells [165].

In addition to nanotopography, various bioactive nanoparticles, including metal nanoparticles, hydroxyapatite, graphene oxide, and carbon nanotubes, modulate cellular behavior and functions while simultaneously enhancing the mechanical strength of scaffolds [166]. Fu et al. [167] developed L-lysine-functionalized graphene oxide (Lys-g-GO) nanoparticles and mussel-inspired AuNPs (AuNPs-PDA), which are coated onto PLGA scaffolds to improve their biological functionality. The resulting AuNPs-PDA@PLGA/Lys-g-GO composite scaffolds exhibited a 3D interconnecting channel-like pore structure with honeycomb-like microvoids. These scaffolds demonstrated excellent mechanical strength, hydrophilicity, and antibacterial properties, significantly improving osteoblast adhesion, proliferation, osteogenic differentiation, and calcification in vitro. In vivo studies revealed significant promotion of new bone formation and collagen deposition at a rabbit radial defect site, confirming excellent biocompatibility [167]. The mechanical, biochemical, topographical, and electrical properties of scaffolds directly influence neural cell behavior in neural tissue engineering. PLGA polymeric scaffolds with narrow-grooved topography stimulate neuronal development and guidance [168]. Razavi et al. [169] further demonstrated that electrospun PLGA nanofibrous conduits with biofunctionalized inner surfaces containing laminin, brain-derived neurotrophic factor (BDNF), and AuNPs in chitosan nanoparticles (CNPs) exhibited nerve regeneration capabilities compared to autografts, which presents a promising solution to alleviate the challenge of insufficient autograft resources. These laminin-coated PLGA conduits with BDNF-CNPs and AuNPs-CNPs, seeded with rat-adipose-derived stem cells (rADSCs) showed a significantly increased expression of Schwann marker (S100) and myelin marker (MBP), which suggested that rADSCs were differentiated into Schwann-like cells with improved neuron diameter and an increased number and thickness of myelin sheath for axonal regeneration in peripheral nerves and other neurological repair applications.

Analogously, multifunctional nanofibrous scaffolds were fabricated using the biodegradable PLGA nanofibers and core–shell microspheres composed of a highly branched AuNP core and folic acid (FA)-conjugated chitosan (CS) polymeric shell that is embedded with surface-enhanced Raman-scattering (SERS) R6G reporter molecules. These scaffolds facilitate tissue regeneration while enabling postoperative cancer monitoring and therapy by targeting and killing residual cancer cells [170]. Similarly, Aydeger et al. [171] developed hybrid micro/nano-channeled film scaffolds using PCL/PLGA, which were surface-decorated with IKVAV (Ile-Lys-Val-Ala-Val) pentapeptide and AuNPs (PCL/PLGA-AuNPs-IKVAV) on their surfaces. These scaffolds with channeled groups decorated with AuNPs significantly enhanced long-distance axonal regeneration through neurite outgrowth and neuronal differentiation along linear lines when coupled with electrical stimulation, compared to polypyrrole (PPy)-coated scaffolds. Customizable 3D bioprinting bioinks have also been developed for tissue-specific applications, utilizing biocompatible and mechanically tunable properties of gelatin methacrylolyl (GelMA) bioinks. GelMa bioink, with varying polymer concentrations and crosslinking time, incorporated with spherical AuNPs (50 nm in diameter) or 2D transition metal carbide (MXene; titanium carbide; Ti_3_C_2_T_x_) nanosheets enhanced biological properties, printability, and exhibited improved rheological properties, and also increased the conductivity of the 3D conductive tissue construct. The optimal conditions for the cellular elongation and spreading of skeletal muscle C2C12 cells were achieved with 2% GelMA crosslinked for 4 min. Furthermore, the inherent conductive properties of AuNPs and MXene nanosheets facilitated C2C12 differentiation even in the absence of external electrical stimulation [172].

In addition to biomaterial topography, electrical and magnetic stimuli serve as important therapeutic approaches in tissue regeneration and regenerative medicine. A 2D platform coated with polyethyleneimine (PEI) and AuNPs (39 nm) induced the neurite elongation in PC12 cells. Following pulsed electrical stimulation (100 mV/mm) of PC12 cultured on the AuNP-coated surface, neurites extended to a length of 120 ± 4 µm, a substantial increase compared to 15 ± 3 µm in unstimulated cells, demonstrating the synergistic effects of AuNPs and electrical stimulation on neuronal growth [173]. Recent advances in regenerative medicine, particularly in nanomedicine, have highlighted the potential of metal nanoparticle-loaded or embedded scaffolds/conduits to induce topographical modifications, enhance neurotrophic factor secretion, improve ion flow, and regulate electrical signals in nerve regeneration [174]. The anti-inflammatory properties of AuNPs, mediated through reducing the release of Ca^2+^ ions and free radical scavenging, further contribute to nerve tissue regeneration. Saderi et al. [175] fabricated AuNP-doped PCL/chitosan nanofibrous conductive scaffolds (diameter = 114–180 nm) with tunable chitosan concentrations. These scaffolds exhibited improved Schwann cell attachment and proliferation, demonstrating promising potential for peripheral nerve regeneration.

**Figure 7:**
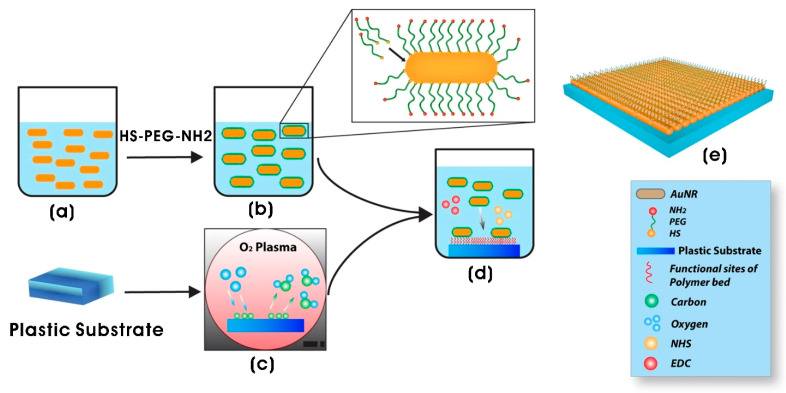
The general procedure for the preparation of functionalized gold nanorod (AuNR) nanocomposite system. (**a**) The synthesis of AuNRs; (**b**) the functionalization of AuNRs; (**c**) oxygen plasma treatment to the plastic Thermanox substrate; (**d**) the assembly of the functionalized AuNRs onto the plastic Thermanox substrate; (**e**) washing with deionized water, followed by treatment with ethanol and ultraviolet (UV) light for sterilization. Reproduced with permission from [182]. Copyright 2017, Nature Publishing Group.

Similarly, manganese (Mn) is an essential trace element required for normal growth development and cellular homeostasis. Particularly, Mn serves as a cofactor for various enzymes involved in neuronal and glial cell functions, as well as in neurotransmitter synthesis and metabolism [176,177]. Interestingly, increased concentrations of manganese (Mn) have been observed to enhance cellular neurodevelopment; however, this enhancement is often accompanied by increased toxicity. To mitigate the cytotoxic effects of certain materials while simultaneously promoting cellular adhesion and conductivity, AuNPs have been incorporated with other materials in the form of composites for peripheral nerve (PN) regeneration. For instance, Bhang et al. [178] demonstrated that AuNPs doped with manganese (MnAuNPs, 8.6 ± 1.4 nm) effectively increased nerve outgrowth by mitigating apoptosis and necrosis in PN regeneration. Moreover, MnAuNPs facilitated the pH-responsive release of Mn^2+^ ions, with a release efficiency of up to 90% at pH 4.0 compared to bare manganese to enhance the differentiation rate of PC12 cells. Conversely, a neutral pH environment reduced Mn^2+^ ion release, leading to decreased cell differentiation rates. AuNPs have also been identified as effective superconductors that positively influence neurite outgrowth. Nerve guidance conduits exhibiting biocompatible, biodegradable, and electrically conductive properties represent a promising approach in peripheral nerve tissue engineering [179]. A 3D multilayer-molded AuNP/polycaprolactone (PCL) nanocomposite nerve conduit, coated with polydopamine (PDA), was fabricated using a 3D printer. These PDA-gold/PCL nanoscaffolds characterized with multiporous structure significantly enhanced angiogenesis, the expression of neurotrophic factors, and the proliferation, adhesion, and neural differentiation of rat bone marrow-derived stem cells (BMSCs) and Schwann cells (SCs) in a mouse model of sciatic nerve injury compared to a conduit without AuNPs. Immunohistochemical analysis confirmed enhanced BMSC differentiation into neurons, as evidenced by an increased expression of S100 and Nestin. Additionally, the thickness and number of myelinated fibers were significantly improved with the AuNP-containing PCL conduit, likely due to its superior nerve regeneration capacity. The porous composite channel likely facilitated cell adhesion and the growth of BMSCs and SCs [180]. A similar phenomenon of enhanced cell adhesion and growth was observed by Wang et al. [181] in neuron-like rat phaeochromocytoma (PC12) cells cultured on porous, conductive poly(3,4-ethylenedioxythiophene)/chitosan/gelatin nanoscaffolds. In the subsequent study, a two-dimensional (2D) nanocomposite composed of gold nanorods (AuNRs; 12 nm diameter; 36 nm length) and polyethylene glycol (PEG) (AuNRs-SH-PEG-NH2) demonstrated significant potential for peripheral nerve regeneration by increasing the differentiation of adipose-derived stem cells (ADSCs) into neural-like cells. This differentiation was confirmed by the upregulation of vimentin, S100β, and glial fibrillary acidic protein (GFAP) expression, suggesting that the presence of AuNRs accelerated neural differentiation. An additional advantage of AuNR-based 3D composite systems is their capacity for surface plasmon resonance activation using infrared (IR) laser sources. This activation can potentially enhance neurogenesis by modulating Ca^2+^ ion channel activity, thereby promoting cell growth and neural differentiation [182,183]. This strategy underscores the importance of designing and developing 3D scaffolds by incorporating AuNRs with optimized surface properties for effective nerve regeneration (Figure 7).

**Figure 8:**
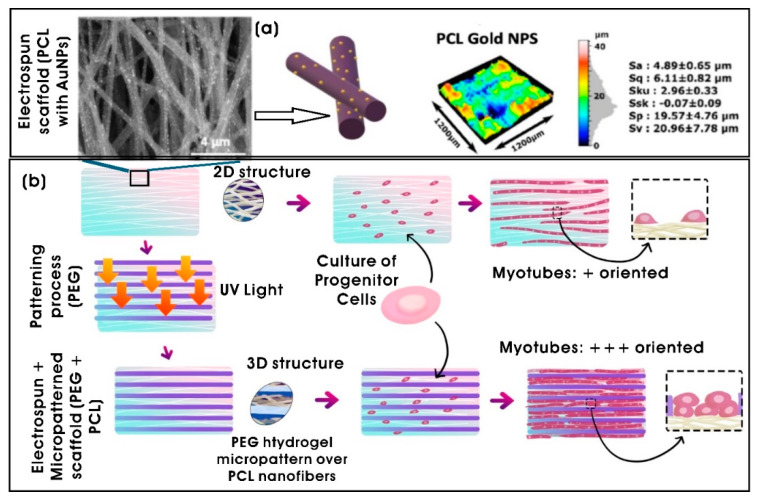
(**a**) SEM images of electrospun fibers coated with gold nanoparticles (AuNPs), and the topography of PCL scaffolds coated with AuNPs. Surface characterization of the PCL scaffolds coated with AuNPs was performed using the Sensofar Confocal profiler. The surface roughness was evaluated using height parameters, including surface valley depth (Sv), surface peak height (Sp), arithmetical mean height (Sa), and root mean square height (Sq). Skewness (Ssk) represents the degree of symmetry of the surface heights relative to the mean plane, while kurtosis (Sku) indicates the sharpness and randomness of the surface features. (**b**) A schematic diagram illustrating the micropatterning of PEG hydrogel on an electrospun PCL mat functionalized with AuNPs, designed to enhance cell guidance and promote myogenic differentiation of C2C12 myoblasts for tissue-engineered skeletal muscle. Reproduced with permission from [189]. Copyright 2021, MDPI Publishers.

Generally, the differentiation of different progenitor cells and stem cells is largely influenced by signals from the surrounding microenvironment. These signals include specific soluble biochemical factors and biophysical signals including surface topography, mechanical properties, roughness, and electrical signals [54]. In particular, the neural differentiation of stem cells is governed by the synergistic effects of surface topography and electrical signals, which hold great promise for the treatment of neurodegenerative diseases. Baranes et al. [184] demonstrated that AuNPs decorated on the surface of the polymeric electrospun nanofibers of 3D PCL/gelatin scaffolds (average fiber diameter of 260 ± 70 nm) significantly promoted neuronal differentiation and maturation. Notably, there was a 2-fold increase in neurite length and a 1.2-fold increase in neurite branching with the uniformly deposited 10 nm AuNPs on the fibers of the scaffolds. The decoration of AuNPs on nanofiber surfaces provides anchorage sites, additional topography cues, and enhanced electrical conductivity, which promotes enhanced morphogenesis, axon elongation, and increased expression of neuronal markers [185]. The functionalization of scaffold surfaces with AuNPs can bring a conducive microenvironment for cellular adhesion and proliferation while also reducing apoptosis and inflammation, thereby improving scar tissue quality [186,187]. Viveros-Moreno et al. [16] evaluated the biocompatibility of alginate–chitosan (Alg/Cs) scaffolds functionalized with spherical AuNPs (~32 nm) and alginate-coated AuNPs. The scaffolds with AuNPs demonstrated regenerative therapeutic potential by modulating inflammatory responses, stimulating fibroblast proliferation, and enhancing the production of collagen fiber and blood vessel formation [188]. Integrating microtopography with a nanofibrous architecture closely replicates the structural features of natural muscle bundles, thereby effectively promoting the formation of aligned myotubes. To achieve controlled cell alignment and elongation in tissue engineering constructs, a multiscale scaffold (PCL-Au-PEG) with a hybrid structure was developed. This scaffold was fabricated using the electrospun PCL nanofibers functionalized with AuNPs and subsequently linearly micropatterned using photolithography with polyethylene glycol (PEG) hydrogel lines. The resulting scaffolds facilitated parallel alignment and efficient differentiation of C2C12 skeletal myoblasts, with increased myotube size observed at a micropatterning space of 500 µm [189] (Figure 8).

**Table 2:** Biomaterial and nanocomposite with different nanotopographical features and their biological applications in tissue engineering and regenerative medicine.

Biomaterial/Nanocomposite	Nanotopographical Features	Biological Applications	Reference
**Organic nanomaterial-modified topographies**			
hAME-encapsulated dextran/chitosan nanoparticles decorated on the polycaprolactone (PCL) nanofibrous scaffold–PVA-based hydrogel	Dextran/chitosan; spherical (d = 212 nm)	Corneal transplantation	[92]
Berberine-loaded chitosan nanoparticles (BerNChs) in alginate-chitosan (Alg-Ch) hydrogel (Alg-Ch/BerNChs)	Spherical; chitosan NPs (NCh) (d = 214 ± 42 nm); BerNChs (d = 252 nm)	Regeneration of injured spinal cord using endometrial stem cells and neural tissue engineering	[93]
Chitosan scaffolds with chitosan and ZnO nanoparticles	Spherical; chitosan NPs (d = 11.98 nm); ZnO NPs (d = 20 nm)	Tissue engineering and regenerative medicine	[94]
Collagen–nanohydroxyapatite (coll-nHAp) scaffolds with miR-26a NPs complexed with cell-penetrating peptide (RALA)	miR-26a cell-penetrating peptide (RALA) nanoparticle; spherical (z-average size: <200 nm up to 40 °C)	Critical-sized calvarial bone defect repair and bone tissue engineering	[102]
PCL–gelatin/collagen nanofibers or collagen nanoparticles	PCL–gelatin/collagen nanofiber (121 ± 28 nm); PCL–gelatin/collagen nanoparticles (141 ± 52 nm)	Skin tissue engineering and regenerative medicine	[103]
Bacterial cellulose hydrogel coated with polydopamine (PDA) micro/nanospheres	Polydopamine (PDA) micro-/nanospheres; spherical (0.65 ± 0.14 µm)	Skin tissue engineering and regenerative medicine	[104]
Porous zirconia scaffold coated with calcium phosphate and loaded with gentamicin-encapsulated PLGA nanoparticles	Gentamicin-loaded PLGA nanoparticles; spherical; #empty PLGA NPs (100–320 nm; average d = 214.6 ± 14 nm); gentamicin-loaded NPs (120–340 nm)	Bone tissue repair and Bone tissue engineering	[108]
PCL microfibrous scaffold with co-sprayed collagen and PPy NPs	Polypyrrole nanoparticles (PPy NPs); spherical (~70 nm)	Neural tissue engineering	[109]
PPy NP-embedded collagen–HAMA hybrid hydrogel	PPy NPs; spherical (d = 40–50 nm)	Spinal cord injury (SCI) repair and neural tissue engineering	[110]
CA-LNP-loaded SiO_2_-doped tricalcium phosphate (TCP) scaffold	Carvacrol-loaded lipid nanoparticles (CA-LNPs); spherical (~129 nm)	Bone tissue engineering	[111]
DS3000 and poly(ethylene glycol)diacrylate (PEG-DA) 3D-printed scaffolds with CNC coating	Cellulose nanocrystals (CNCs) (length × width; 100–200 nm × 5–20 nm)	Tissue engineering and regenerative medicine	[114]
**Inorganic nanomaterial-modified topographies**			
PCL/PLA scaffold by 3D printing technique	Strontium doped bredigite nanoparticles (Bre-Sr); spherical (<200 nm)	Bone tissue engineering	[11]
Poly-L/D-lactide (PLDLA) copolymer scaffold containing BTNP	Al_2_O_3_- and SiO_2_-coated barium titanate nanoparticles (BTNPs); 50–80 nm (round, oval, and angular-shaped)	Bone tissue engineering	[120]
MgO-xAg nanocomposite	MgO (34.2 nm); nano-lamellae MgO-10Cu (101.2 nm length × 9.6 nm thickness)	Bone tissue engineering	[117]
Poly(L-lactic acid) (PLLA) scaffold with GO@SiO2	SiO_2_ nanoparticles on graphene oxide nanosheets (GO@SiO_2_)	Bone tissue engineering	[121]
Glass surface	Cluster-assembled ns-TiO_2_; 20.2 ± 0.5 nm rms roughness (50 nm film thickness) and 29.1 ± 1.0 nm rms roughness (200 nm film thickness)	Nerve regeneration	[159]
Gelatin/Fe_3_O_4_ composite scaffold	Fe_3_O_4_-citrate nanoparticles; flower-like shape (average size of 29.6 ± 3.9 nm)	Cancer therapy and adipose tissue regeneration	[152]
PLLA fibrous scaffold grafted with SPIONs	Superparamagnetic IONPs (SPIONs); average number of aggregates of 6 ± 4 per 100 µm^2^, and the average size of the aggregates was 0.52 ± 0.54 µm^2^	Axonal regeneration and neural tissue engineering	[153]
Silk–gelatin (SG)/polylactic acid (PLA) bilayer nanocomposite	Nerve growth factor-incorporated IONP–graphene nanoparticles (GFPN)-PEI; Fe_3_O_4_ NPs (5–10 nm) distributed on the sheet-like structural rGO	Nerve regeneration and next-generation nerve conduits	[154]
Collagen film with PEG-capped paramagnetic IONPs (collagen/PEG@IOPs film)	PEG@IONPs; Spherical (80 nm in diameter)	Tissue engineering and regenerative medicine	[155]
Alginate/magnetic short nanofiber composite hydrogel	Superparamagnetic Fe_3_O_4_ nanoparticles (SPION); M.SNF of 222 ± 64 nm containing spherical SPIONs (10 ± 2 nm)	Nerve regeneration	[157]
Chitosan-ZnO NPs composite conduit (CZON)	ZnO NPs; spherical (30 nm)	Nerve regeneration and nerve conduits	[142]
PCL/ZnO NPs scaffold	ZnO NPs; spherical (30–80 nm)	Nerve regeneration and nerve conduits	[143]
Poly-ɛ-caprolactone composite nanofibers with ZnO NPs coated with PDA and QK peptides (PCL@Z/P/QK)	ZnO NPs	Orthopedic implants and bone repair and bone tissue engineering	[144]
Gelatin/nanoceria nanocomposite fibers	Nanoceria (CeO_2_ nanoparticles); nanoparticle dispersion (d < 5 nm)	Neuronal tissue engineering and regenerative medicine	[149]
PLA microfiber with streptomycin-loaded hydroxyapatite nanoparticles	Nanohydroxyapatite (nHAp); nanorod (diameter × length; 20–50 nm × 50–150 nm)	Tissue engineering and regenerative medicine	[125]
Chitosan-crosslinked polyaniline nanonets coated with titanium nanotubes (TiO_2_NTs-PANI@CS)	TiO_2_ NTs; TiO_2_ NTs (tube wall thickness × length × inner diameter; 13.84 × 1.15 × 85.07 nm)	Bone tissue engineering	[161]
Hydroxyapatite/Polycaprolactone nanoparticles (HAp/PCL NPs)	Hydroxyapatite NPs; rod-like morphology (length × width; 92.18 ± 18.21 nm × 30.3 ± 4.7 nm)	Bone tissue engineering	[124]
Poly-ɛ-caprolactone (PCL) nanofibrous membrane loaded with tantalum (Ta)/whitlockite (WH) nanoparticles (PCL/Ta/WH)	Tantalum (Ta) (Ta NPs; 50 nm) and whitlockite (WH) nanoparticles	Bone repair and tissue engineering	[129]
PCL/nanoparticulate willemite (npW) composite scaffold (3D printing)	Silicium dioxide (SiO_2_) (d = 5–20 nm); zinc oxide (ZnO) (<100 nm); willemite (Zn_2_SiO_4_) nanoparticle (spherical, d = 20–70 nm)	Osteonecrosis defects and steroid-associated osteonecrosis	[136]
Glass surface coated with Ag, Au, or ZnO NPs	AgNPs or AuNPs or ZnO NPs; particle diameters (AgNPs: 110 ± 40 nm; AuNPs: 100 ± 40 nm; ZnONPs: 115 ± 45 nm)	Nerve regeneration and regenerative medicine	[163]
PEG hydrogel/Ag nanowire composite micropattern-based sensor on a flexible PET film	Ag nanowires on polyethylene terephthalate (PET) film	Neural implant or graft and neural stem cell therapy	[165]
AuNPs-PDA@PLGA/Lys-g-GO composite scaffolds	L-lysine-grafted GO (Lys-g-GO) (wrinkled surface) NPs and AuNP–Polydopamine (AuNPs-PDA) NPs	Bone defect treatment and bone tissue engineering	[167]
PLGA nanofibrous conduit functionalized with laminin containing BDNF and AuNPs-CNPs	BDNF- and AuNP-encapsulated chitosan nanoparticles (BDNF/AuNPs-CNPs); BDNF/AuNP-encapsulated CNPs (d = 77.8 ± 2.05 µm)	Nerve regeneration and neurological repair	[169]
Theranostic AuNP-encapsulated PLGA microspheres embedded in the nanofibrous structure	Gold nanoparticle (AuNP) core, a conjugated folic acid (FA)–chitosan (CS) polymeric shell; spherical AuNPs (d = 60–110 nm)	Tissue regeneration and postoperative cancer management	[170]
Micro/nano-channeled PCL/PLGA film scaffold surface decorated with IKVAV peptide/AuNPs	Surface decorated with IKVAV pentapeptide/AuNPs; spherical AuNPs (d = 50 nm)	Nerve regeneration and neural tissue engineering	[171]
Gelatin methacrylolyl (GelMa) with MXene or AuNPs nanocomposite conductive bioink for 3D printing	AuNPs and MXene (titanium carbide; Ti_3_C_2_T_x_) nanosheets; MXene (Ti_3_C_2_Tx) nanosheets (lateral size × thickness; 2–3 µm × 3–4 nm (3–4 layers); spherical AuNPs (d = 50 nm)	Skeletal muscle tissue engineering	[172]
Polyethyleneimine (PEI)-coated cover glass with adsorbed gold nanoparticles (GNPs)	AuNPs; spherical (d = 39 nm)	Nerve regeneration and neural tissue engineering	[173]
PCL/chitosan nanofibers (d = 114–180 nm with varying chitosan concentrations)	AuNPs; spherical (d = 175 ± 69 nm)	Peripheral nerve regeneration and neural tissue engineering	[175]
PDA-gold/PCL nanocomposite channels (nerve conduit)	AuNPs and polydopamine	Peripheral nerve regeneration and neural tissue engineering	[180]
AuNR-functionalized polyethylene glycol (PEG) (AuNRs-SH-PEG-NH2) composite (2D) system	Gold nanorods (AuNRs); AuNRs (12 nm diameter; 36 nm length)	Neural tissue engineering	[182]
PCL/gelatin nanofiber scaffolds (average fiber diameter of 260 ± 70 nm)	AuNPs (d = 10 nm)	Neuronal tissue engineering and neural regeneration	[184]
Alginate–chitosan (Alg/Cs) scaffolds with AuNPs	AuNPs; spherical (d = ~32 nm)	Tissue engineering and regenerative medicine	[16]
PCL-AuNPs and PCL-Au-PEG 3D scaffolds	AuNPs; spherical (d = 15.65 ± 6.41 nm)	Skeletal muscle tissue engineering	[189]

## 6. Conclusions and Future Perspectives

In conclusion, nanotopographical modifications on polymeric nanocomposite scaffolds play a pivotal role in modulating scaffold topography, cellular responses, and regenerative outcomes, making them indispensable for next-generation tissue engineering strategies. Despite substantial evidence demonstrating the effectiveness of nanotopographical features in enhancing scaffold biocompatibility and promoting tissue regeneration, the underlying mechanisms governing these interactions remain incompletely understood. Many studies provide qualitative insights, while some yield conflicting results. Therefore, rigorous investigations with standardized protocols, well-controlled experimental conditions, and scientific repetitions are imperative to elucidate the precise effects of nanotopography on cellular and tissue responses. Further advancements in nanomaterial engineering are necessary to develop improved nanomaterials with tunable dimensions and incorporation techniques that enable precise patterning across multiple length scales. Achieving uniform nanomaterial distribution on polymeric nanocomposite scaffold surfaces and within scaffold architecture remains a challenge, requiring innovative fabricating methodologies. In electrospun fiber-based scaffolds, scalable and reproducible techniques for fabricating nanofibrous structures with controlled orientation must be developed to enhance their translational potential. Additionally, the interactions between nanomaterials of various morphologies and dimensions with biological entities should be extensively evaluated, both in vitro and in vivo, to ensure their suitability for biomedical applications, including tissue engineering, bioelectronics, and implantable devices.

Another critical consideration is the long-term stability of biomaterials incorporating inorganic materials, as well as their biodistribution, circulation time, clearance mechanisms, and potential cytotoxicity. There is also a need for comprehensive in vivo studies to assess these factors across different cell types and physiological environments to ensure their safety and efficacy. The successful development of reliable nanotopographical polymeric nanocomposite scaffolds through simple strategies could potentially reduce reliance on expensive and complex nanofabrication techniques, such as photolithography, electro-beam lithography, nanoimprint lithography, and various etching methods. Ultimately, bridging the gap between laboratory research and clinical application requires a multidisciplinary approach that integrates advanced fabrication strategies, rigorous biological validation, and scalable manufacturing processes. These efforts will be instrumental in translating nanotopographical scaffold technologies into commercially viable biomedical products for use in tissue engineering, regenerative medicine, and other biomedical fields.
